# Biomarkers for Pancreatic Neuroendocrine Neoplasms (PanNENs) Management—An Updated Review

**DOI:** 10.3389/fonc.2020.00831

**Published:** 2020-05-27

**Authors:** Martine Bocchini, Fabio Nicolini, Stefano Severi, Alberto Bongiovanni, Toni Ibrahim, Giorgia Simonetti, Ilaria Grassi, Massimiliano Mazza

**Affiliations:** ^1^Biosciences Laboratory, Istituto Scientifico Romagnolo per lo Studio e la Cura dei Tumori (IRST) IRCCS, Meldola, Italy; ^2^Nuclear Medicine and Radiometabolic Units, Istituto Scientifico Romagnolo per lo Studio e la Cura dei Tumori (IRST) IRCCS, Meldola, Italy; ^3^Osteoncology and Rare Tumors Center, Istituto Scientifico Romagnolo per lo Studio e la Cura dei Tumori (IRST) IRCCS, Meldola, Italy

**Keywords:** pancreatic tumor, pancreatic neuroendocrine tumor, biomarker, neuroendocrine syndrome, FDG (18F-fluorodeoxyglucose)-PET/CT

## Abstract

Pancreatic neuroendocrine tumors (PanNENs) are rare sporadic cancers or develop as part of hereditary syndromes. PanNENs can be both functioning and non-functioning based on whether they produce bioactive peptides. Some PanNENs are well differentiated while others—poorly. Symptoms, thus, depend on both oncological and hormonal causes. PanNEN diagnosis and treatment benefit from and in some instances are guided by biomarker monitoring. However, plasmatic monoanalytes are only suggestive of PanNEN pathological status and their positivity is typically followed by deepen diagnostic analyses through imaging techniques. There is a strong need for new biomarkers and follow-up modalities aimed to improve the outcome of PanNEN patients. Liquid biopsy follow-up, i.e., sequential analysis on tumor biomarkers in body fluids offers a great potential, that need to be substantiated by additional studies focusing on the specific markers and the timing of the analyses. This review provides the most updated panorama on PanNEN biomarkers.

## Introduction

Neuroendocrine neoplasms (NENs) are rare and heterogeneous tumors of epithelial origin arising from cells of the neuroendocrine system. Pancreatic NENs (PanNENs) are low incidence diseases accounting for less than 3% of all pancreatic malignancies but their prevalence is relatively high and is actually rising (1). PanNEN patients account for 8.1% of total NEN cases (SEER 18) (2), present metastases at diagnosis in 60–80% of cases (3) and can be subgrouped in functioning (F-PanNENs) and non-functioning neoplasms (NF-PanNENs) depending on their ability to secrete active hormones associated with a specific symptomatology. They can occur as sporadic and isolated tumors or in the context of complex hereditary syndromes, such as multiple endocrine neoplasia type 1 (MEN1), von Hippel–Lindau disease (VHL), neurofibromatosis 1, and tuberous sclerosis (4–6). MEN1, in particular, is the commonest syndrome associated with PanNENs and about 10% of all PanNEN patients are affected by MEN1 syndrome (1, 7). PanNENs prognosis differs widely, with some tumors having an indolent nature, with a reasonable length of survival even with a metastatic presentation and others being very aggressive with poor prognosis. PanNENs prognosis heterogeneity is in part recognized by the World Health Organization (WHO) classification system. Three independent PanNEN staging systems coexist and are suggested by the European Neuroendocrine Tumor Society (ENETS), the American Joint Committee on Cancer (AJCC) and the World Health Organization (WHO) respectively (2, 8–10). WHO classification is based on cellular proliferation (measured as mitotic count and Ki-67 expression; see Table 1). WHO has recently updated NENs classification whereby well-differentiated NENs are defined Neuroendocrine Tumors (NETs) regardless the grading. This has generated a novel subgroup of well-differentiated tumors with high Ki-67/mitotic index as G3 and poorly differentiated NENs defined as Neuroendocrine Carcinomas (NEC) which are G3 by definition (2, 8, 10). The ENETS staging system is based on TNM classification (1, 14) whereas the AJCC—draws on the TNM staging for pancreatic adenocarcinoma (5, 9); see Table 1 for a comparison). Although the grade of disease is prognostic, several differences in the clinical behavior remain between each subgroup, making personalized treatment challenging for PanNENs. There is a clear unmet clinical need for novel prognostic and predictive biomarkers able to improve grading and staging assessments, guide prognostication and support treatment decisions. We will provide here a general overview of the existing and promising prognostic and predictive biomarkers for PanNENs.

**Table 1:** Current WHO grading guidelines and 8th AJCC/UICC—ENETS consensus for pancreatic neuroendocrine neoplasms (11, 12).

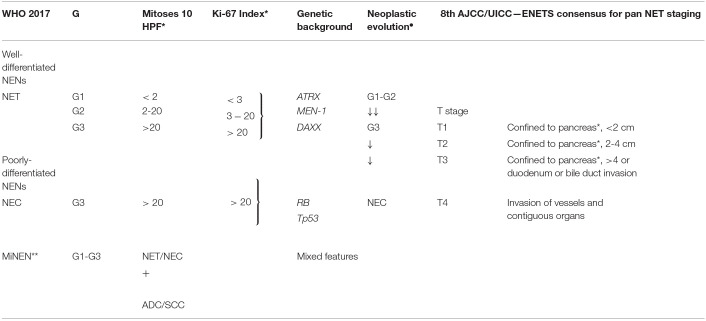

WHO, World Health Organization; NEN, Neuroendocrine Neoplasm; NET/C, Neuroendocrine Tumour/Carcinoma; HPF, Hight Power Field; AJCC, American Joint Committee on Cancer; ENETS, European Neuroendocrine Tumour Society; UICC, Union for International Cancer Control; ADC, Adenocarcinoma; SCC, Squamous cell Carcinoma. ^∙^Neoplastic evolution Current classification considers the possibility of an evolution with time of a well-differentiated G1-G2 NEN to a higher G3 and, even more rarely, toward a poorly differentiated NEC (13). **MiNENs (Mixed-NENs): may contain of non- neuroendocrine components (e.g., adeno or squamous) and neuroendocrine ones (at least 30% for each component) (11). *Specific parameters for PanNET according to 8th AJCC/UICC-ENETS consensus.

## Pancreatic Nens Biomarkers

Correct diagnosis and accurate staging are of primary importance when treating cancer patients and the use of biomarkers is pivotal in this challenge. An ideal biomarker should display high sensitivity for the diagnosis of NENs, to predict tumor aggressiveness (prognostic biomarker) and/or response to treatment (predictive biomarker) (15). Since several factors impact NEN patients' survival, a multi-analyte approach, which takes into consideration clinical, biochemical, histological and molecular features of the disease is required (16). Several parameters correlate with the overall survival of NEN patients. They include tumor localization, size, grade and stage, vascularization, presence of necrotic tissue and the presence of metastases (17, 18). NEN diagnosis starts with the biochemical quantification of circulating analytes in the plasma and/or serum of patients. Neuroendocrine markers can be divided into two main groups: non-specific markers that are virtually produced by all NENs (19) and specific markers that are primarily produced by F-NENs (Table 2).

**Table 2:** Biochemical biomarkers in use for PanNEN diagnosis, prognosis, and treatment monitoring.

**Biochemical markers**			**Source**	**Level**	**Sens. (%)**	**Spec. (%)**	**Combinations improving sens./spec**.	**Clinical use**	**References**
Non–specific	Chromogranin A	CHGA	Serum	63–14.750 ug/l	60–83	72–85	NSE; PP	For diagnosis and follow up in GEP-NENs and treatment monitoring	(20, 21) (22, 23)
	Neuron-specific enolase	NSE	Plasma	5–92 ug/l	33	73	CHGA	For diagnosis and follow up in GEP-NENs and treatment monitoring	(20, 21) (22, 23)
	Pancreatic-Polipetide	PP	Plasma	480–780 pg/ml	31-63	67	CHGA	For diagnosis and follow up in PanNENs	(23)
	Human Corionic Gonadotropin	HCG	Serum	Increased	na	Na	AFP; CHGA; PP; HCG	Indicative of pancreatic origin	(24)
	Alpha Fetoprotein	AFP	Serum	Increased	na	Na	HCG; CHGA; PP	Indicative of pancreatic origin and de-differentiation	(25, 26)
Specific	Gastrin	GAS	Serum	≥300 pg/mL	94	100	MEN-1; ZES	Diagnostic for Gastrinoma of pancreatic origin	(24, 27)
	Insulin	INS	Serum/ Plasma	≥43^∙^ pmol/L	52 - 94	92−100	Whipple's triad	Diagnostic for Insulinoma; suggesting for WD NETs.	(28)
	Glucagon	GCG	Plasma	500–1000 pg/mL	High	High	-	Diagnostic for Glucagonoma; suggesting for WD NETs; Indication for liver metastases	(24)
	Somatostatin	SST	Plasma	Increased°	na	Low	SSoma syndrome°	Diagnostic for SSoma of pancreatic origin;	(24)
	Vasoactive Intestinal Peptide	VIP	Serum/Plasma	75^∙^−200 pg/dL	na	na	Verner Morrison	Diagnostic for ViPoma of pancreatic tail origin.	(29)

*PanNENs, Pancreatic Neuroendocrine Neoplasia; GEP-NENs, Gastro-Entero-Pancreatic Neoplasia; WD NETs, well differentiated tumors; Sens., sensibility; Spec., specificity. ^∙^Diagnostic serum/plasma level in association with specific syndrome. °Somatostatin increase is very a-specific, increase SS level with SSoma syndrome is suggesting for GEP-NENs*.

### Pancreatic NENs Non–specific Biomarkers

**Figure 1:**
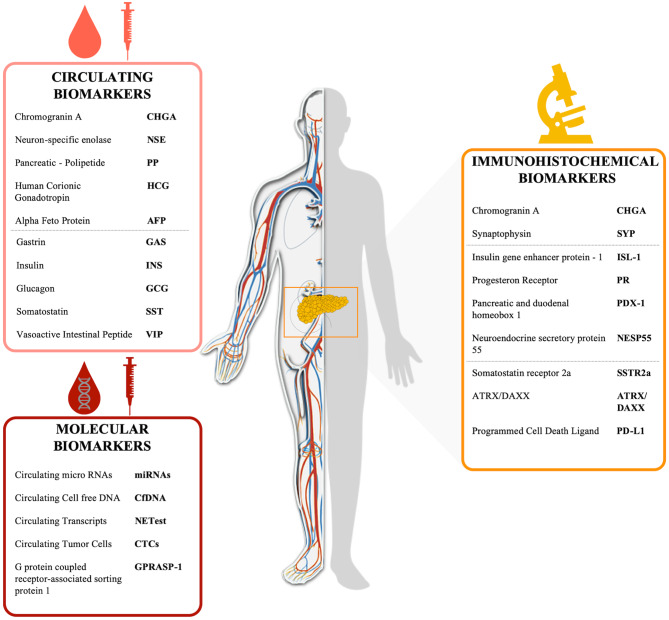
Schematic representation of PanNEN biomarkers Circulating peptides (i.e., CHGA, PP) are circled in light red, markers assayed by IHC on tissue (i.e., CHGA, SSTR) are circled in yellow and circulating molecular biomarkers (i.e., RNA transcripts, cfDNA) are circled in red.

Non-specific PanNEN biomarkers include chromogranin-A (CHGA), Neuron Specific Enolase (NSE), Pancreatic Polipeptide (PP), Human Chorionic Gonadotropin (HCG), and Alpha Fetoprotein (AFP) (Table 2, Figure 1). Biochemical evaluation of these analytes can be easily performed on serum/plasma fraction of patients with suspected NENs. Aberrant levels of such non-specific markers should drive further and deepen diagnostic tests (30).

**Chromogranin-A (CHGA)** is a glycoprotein secreted by neurons and neuroendocrine cells, which is a precursor of bioactive substances such as pancreastatin, catestatin and vastatins I and II (31). Despite all members of granin family can be secreted by neuroendocrine tumors, CHGA is the only one routinely used in clinical practice. The assay has a high sensitivity (32) and good specificity (19). Increased levels of CHGA can be detected both in plasma and serum with a good correlation, suggesting either measurement can provide reliable evaluations of circulating CHGA (33). Circulating CHGA has been reported to correlate with tumor progression (19), presence of metastases (34), tumor burden and response to treatment in NENs, including PanNENs. In fact, CHGA decrease in serum can be considered a surrogate marker for treatment efficacy (35). In contrast, despite two to three-fold increase of CHGA can be considered marker for NENs and also for neuroendocrine differentiation of other non-neuroendocrine cancers, several non-pathological factors, such as food intake (6) and several non-neoplastic endocrine diseases can increase its level in the bloodstream (36), making diagnosis challenging. For those patients affected by concomitant conditions, CHGA assay specificity may decrease up to 50%. Therefore, CHGA should be never considered a first-line diagnostic or screening tool in these sub-populations (37). Despite the above-mentioned limitations, up to now CHGA is the most used liquid biomarker not only in the diagnosis but also during the follow-up of NEN patients.

**Neuron Specific Enolase (NSE)** is an enzyme found in neurons and neuroendocrine cells, even if only 30–50%, of NENs secretes NSE (22, 32, 38). This marker may be elevated in 38–40% of high-grade GEP-NENs, including PanNENs thus providing also prognostic information (39). NSE levels have been directly associated with tumor differentiation, aggressiveness and size (39, 40) and it was found to inversely correlate with overall survival (OS) and with progression-free survival (PFS) in ENETS TNM stage IV. NSE has low sensibility but relatively high specificity (see Table 2). Indeed, NSE can be virtually overexpressed also by several non-neuroendocrine tumors, such as parathyroid cancer, prostate carcinoma, neuroblastoma, and it has been correlated with poor differentiation, prognosis and high-grade disease (24). For these reasons NSE alone is rarely used for diagnostic purposes or to distinguish NENs from non-endocrine tumors. Up to date, there is no robust evidence of the predictive role of NSE in predicting therapy efficacy and monitoring patients during follow-up. On the other hand, elevated baseline CHGA/NSE provide prognostic information on PFS and survival in patients with advanced PanNEN treated with the mTOR inhibitor everolimus (41). Evaluation of both NSE and CHGA concentration increases the reliability of NEN diagnosis; however, given the non-specific nature of these markers, they do not provide information on the primary tumor site and its origin (24).

**Pancreatic Polypeptide (PP)** PP is a 36 amino acid linear oligopeptide, primary secreted by the PP cells of Langerhans' islets (42). Despite its specific role is not well clarified it is supposed to regulate pancreatic, GI secretions (32) and hepatic glycogen levels (38). PP is generally considered a neuroendocrine differentiation marker with good specificity but low and variable sensitivity (30) (Table 2). Since 2015, PP has been suggested for the diagnosis of PanNENs (NCCN guidelines) (43) and ESMO 2012 consensus guidelines already considered PP diagnostic also for NF-PanNENs (29). Despite PP has been observed to be elevated in metastatic disease with increased sensitivity (up to 80%) (44), <50% of PanNEN patients presents with elevated serum PP (19). Additionally, serum concentrations of PP can be increased by many factors, including physical exercise, hypoglycemia, and food intake (32), as well as decreased by somatostatin and hyperglycemia, diarrhea, laxative abuse, increased age, GI inflammatory processes and chronic renal disease (45). Detection of high levels of circulating PP, together with CHGA is suggestive for PanNENs with increased sensitivity (30, 42). Production of PP and/or CHGA is observed in 100% of spontaneous and hereditary gastrinomas (46). In contrast, decline of PP level during patients monitoring is considered a good prognostic marker (19).

Finally, **human chorionic gonadotropin (HCG)** and **alpha-fetoprotein (AFP)** can be also considered in biochemical assessment of certain malignancies, although their use is limited (24). HCG is a glycoprotein physiologically synthesized by syncytiotrophoblastic cells of the placenta during pregnancy (24, 32) and it is composed of α and β subunits. The β subunit (β-HCG) is specific, since tumor cells usually lack the mechanism to link α and β subunits. An increased secretion of the β subunit is reported in pancreatic tumors and PanNENs. AFP is a peptide hormone produced during development. In adults increase of AFP in serum has been reported in NENs (25, 26). AFP-producing PanNENs are rare and often associated with other malignancies (47, 48). However, the literature is controversial on the sensitivity and specificity HCG and AFP, thus limiting their use in NENs (37, 49).

### PanNENs Specific Biomarkers

Bioactive peptides retrieved in the blood of F-PanNEN patients are useful prognostic and predictive biomarkers (24). However, hormones are not always secreted and retrievable from the blood. Indeed, evaluation of expression directly on the neoplastic tissue is the gold standard for diagnosis. In addition, symptoms associated with their increased levels help both to diagnose and to identify the primary site of disease (50). F-PanNENs are named after the hormones they produce as insulinomas, glucagonomas, gastrinomas, somatostatinomas, VIPomas, which are suggestive of their cell-of-origin.

### Circulating Biomarkers

**Gastrin (GAS)** is a linear peptide hormone secreted by G–cells of pyloric antrum, duodenum and pancreas implicated in the regulation of chloride acid release from parietal cells in the stomach, gastric motility and pancreatic secretion. A plasma concentration of GAS >300 pg/mL correlates with the presence of gastrinomas, even if GAS is secreted as well by functioning NENs especially in the context of MEN-1 and Zollinger–Ellison syndrome (ZES) (32).

**Insulin (INS)** is a dimeric peptide hormone of 51 amino acids, physiologically secreted by the β cells of the pancreatic islets in response to glycemia increase and involved in the regulation of body anabolism. INS can increase as a consequence of several oncologic and non-oncologic conditions, therefore, its concentration alone does not represent a solid marker for insulinoma. Insulinoma should be suspected when patients display the so-called “Whipple's triad” symptoms: clinical evidence of hypoglycemia, serum glucose ≤ 40 mg/dL and improvement following administration of glucose (51).

**Glucagon (GCG)** is a peptide hormone secreted by pancreatic α-cells to increase catabolism thereby mobilizing energy reserves to free glucose molecules via gluconeogenesis and glycogenolysis. An increased plasma GCG level >500 pg/mL is indicative of glucagonoma albeit requires further diagnostic work-up to exclude non-oncologic reasons. By contrast, GCG levels >1000 pg/mL are diagnostic for glucagonoma and used in the clinical practice (52).

**Somatostatin (SST**) is a peptide hormone physiologically secreted by pancreatic δ-cells, APUD cells and gastric antrum D cells (53). SST can repress GCG and INS secretion by α and β cells of the pancreas, respectively. SST excess induces non-specific manifestations and it can result in the formation of gallstones, intolerance to fat in the diet, diarrhea and diabetes. Furthermore, increased levels of SST are not only associated with somatostatinoma of the pancreas but also with various extra-Pancreatic NENs (54). Hence, SST level *per se* is not sufficient to diagnose somatostatinoma but it requires very careful clinical assessment.

**Vasoactive Intestinal Peptide (VIP)** is a peptide hormone released by pancreatic and brain cells. It is both a neurotransmitter and a potent vasodilator regulating smooth muscle activity, epithelial cell secretion and blood flow in the gastrointestinal tract. VIPoma, a non-ß pancreatic islet cell tumor, shows a syndrome of watery diarrhea, hypokalemia, and achlorhydria (WDHA syndrome) and it is diagnosed by a serum VIP concentration above 200 pg/dL. A mild increase in VIP concentration (75-200 pg/dL) can be also considered in patients with Verner Morrison syndrome (29). These biomarkers can be suggestive of a PanNEN. However, symptoms can often be nuanced or aspecific, and careful clinical and histo-pathological assessment remains mandatory.

### Tissue Biomarkers

Histological diagnosis is usually assessed on surgical or endoscopic biopsies, on which morphological and marker distribution analysis is performed by immunohistochemistry (IHC) (29) (Table 3). PanNENs can also produce hormones that are not subsequently secreted, and specific stains for GAS, INS, and SST can confirm clinical symptoms without biochemical increase in serum. However, IHC alone for hormones and bioactive peptides cannot prove site of origin and confirm functionality of NENs (29). At present chromogranin A (CHGA) and synaptophysin (SYP) are considered the most specific markers for NEN differentiation by immunohistochemistry (62). CHGA is contained in the granules of neurons and pancreatic cells, it is a precursor of several functional peptide hormones such as vasostatins and pancreastatin. CHGA is widely expressed in well–differentiated NENs whereas generally low or focally positive in poorly–differentiated NEC (55). SYP is an integral transmembrane glycoprotein expressed in neuroendocrine cells and neurons involved in synaptic transmission with a diffuse cytoplasmic immunostaining (63). CHGA and SYP combined assessment represents the first of a multi–step approach currently in use to confirm the neuroendocrine nature of the disease and then its pancreatic origin.

**Table 3:** Immunohistochemical (IHC) biomarkers for PanNENs diagnosis, prognosis and treatment monitoring.

**Immunohistochemical markers (IHC)**			**Source**	**Level**	**Combinations improving sens./spec**.	**Clinical use**	**References**
Differentiation	Chromogranin A	CHGA	Surgical/endoscopic biopsy	Over-expressed	SYP	Diagnosis of NENs; Grading; Differentiation	(23) (24)
	Synaptophysin	SYP	Surgical/endoscopic biopsy	Over-expressed	CHGA	Diagnosis of GEP-NENs; grading; differentiation	(24)
Site of Origin	Insulin gene neanche homeeobox - 1	ISL-2	Surgical/endoscopic biopsy	Over-expressed in endocrine pancreas	Low expression in case of Gastrinoma	Over-expressed in Pan NENs (especially in WD tumors)	(55)
	Progesteron Receptor	PGR	Surgical/endoscopic biopsy	Positive	CHGA + SYP	Indicative of pancreatic origin (40-75%) (negative in GI-NENs)	(56)
	Pancreatic and duodenal homeobox 1	PDX-1	Surgical/endoscopic biopsy	Positive	CHGA + SYP	Indicative of pancreatic origin	(57)
	Neuroendocrine secretory protein 55	NESP55	Surgical/endoscopic biopsy	Focally positive	CHGA + SYP	Indicative of pancreatic origin (40−50%)	(56)
Prognostic/ Predictive	Somatostatin receptors 2a	SSTR2a	Surgical/endoscopic biopsy	Over-expressed	CHGA + SYP	Indicative of pancreatic origin; Predictive for PRRT treatment; inverse correlation with grading.	(58) (59)
	ATRX/DAXX	ATRX/ DAXX	Surgical/endoscopic biopsy	Loss of expression	CHGA + SYP	Prognostic for tumor aggressiveness; (associated with WD tumors)	(60)
	Programmed Cell Death Ligand	PD-L1	Surgical/endoscopic biopsy	Over-expressed	CHGA + SYP	Prognostic/Predictive for anti-PD-L1 therapeutic agents	(61)

*PanNENs, Pancreatic Neuroendocrine Neoplasia; GEP-NENs, Gastro-Entero-Pancreatic Neoplasia; WD NETs, well differentiated tumors*.

## Emerging Markers in PanNEN

### Tissue Biomarkers

Besides the validated diagnostic markers, other tissue biomarkers are under investigation to improve PanNENs management providing information on the site of origin, grading, immune and genetic landscape of the disease. In addition, novel biomarkers could be new therapeutic targets. Up to now several immunohistochemical panels have been proposed to identify primary tumor site of origin, especially in NENs of the gastro-entero-pancreatic (GEP-NENs) tract. Although many recent studies focused on these biomarkers they are not routinely used and validated for diagnosis and/or prognosis in PanNENs management.

**Islet 1 (ISL-1)** is a homeobox transcription factor expressed in all endocrine pancreatic cells (57). This pattern of expression suggests a general role in the development of multiple cell lineages of the endocrine pancreas. ISL-1 expression is detected in 70–82% of panNENs (64). Unfortunately, other GI–NENs, in particular NENs of the rectum, overexpress this marker (65) and gastrinomas of the pancreas show low expression of ISL-1 making its application as a general PanNEN diagnostic biomarker troublesome.

**Progesteron Receptor (PR**), represent a widely—studied, but still incoming and more specific pancreatic marker. Nuclear positivity for PR has been reported in most pancreatic endocrine tumors, and recent studies confirm PR expression in 40–75% of PanNENs (56, 64). In addition, PR immunoreactivity has been demonstrated to be strictly confined to endocrine compartment of normal and neoplastic human pancreatic islets (56, 64) and to be significantly associated with a favorable prognosis and a lower clinical stage (66). The relative expression of PR isoforms (PRA; PRB) have been reported to have a prognostic role in NENs from different site of origin (e.g., breast) (67, 68). Recent findings focused on the role of PRA and PRB in PanNENs demonstrated that PRB activation promotes Cyclin D1 (CCND1) overexpression and, as a consequence of c-Fos and c-Jun induction transcription factors supporting cell proliferation and tumorigenesis (69). In addition, progesterone signaling via PRA could inhibit tumorigenesis by PRB suppression. In addition, PRA can be a suitable predictive factor in PanNEN and inversely correlated with tumor progression (70).

**Neuroendocrine secretory protein 55 (NESP55**) is a protein belonging to the chromogranin family which can be considered highly specific marker for PanNENs, since other GI-NENs subtypes show low to none expression of this protein (64). Recent findings report focal and specific expression of NESP55 in 40–74% of PanNENs in contrast with very rare expression observed in other GI-NENs and NENs of the lung and rectum (5 and 8%, respectively) (64, 71).

**Paired box 8 (PAX 8)** represents a transcription factor able to regulate organogenesis in a variety of organs (72). Although PAX8 has been considered a marker for renal development and neoplasms, Sangoi et al. observed high PAX8 reactivity in PanNENs and normal pancreatic islets in a large tissue microarray evaluation (73). In contrast with ileal or pulmonary NETs and NENs of duodenum, stomach, and rectum which were negative to PAX8 staining or show very low expression, respectively. PAX8 has been demonstrated to be particularly useful in metastatic NENs with unknown primary tumor site, the expression PAX8 in combination with ISL-1 could indicate pancreatic origin (5).

**Pancreatic and duodenal homeobox 1 (PDX-1)** is transcriptional activator of several genes, including insulin, somatostatin, glucokinase, islet amyloid polypeptide, and glucose transporter type 2 (74). PDX-1 immunoreactivity is reported in 54–100% of PanNENs (64). Despite PDX-1 can be expressed also by other GI-NENs, NENs of the ileum have been reported to be negative for PDX1 thus it can be useful, especially when used in combination with ISL-1, PAX8, and/or NESP55 in defining pancreatic site of origin when it is unclear. In addition, PDX-1 is involved in the early development of the pancreas and plays a key role in glucose-dependent regulation of insulin gene expression (74).

Among those, combinations of Islet 1 (ISL-1), Progesteron Receptor (PR), neuroendocrine secretory protein 55 **(**NESP55), paired box 8 (PAX8), and Pancreatic and duodenal homeobox 1 **(**PDX1) suggest pancreatic origin (73, 75–77). In addition, the well-known Somatostatin Receptors (SSTRs) and GLUT-1 are companion markers for imaging techniques which fulfill a primary role in PanNEN diagnosis and prognosis.

**Somatostatin receptors 2a and 5 (SSTR2a and SSTR5)** have been widely studied as prognostic and predictive biomarker in GEP-NENs since most of GEP-NENs shows diffuse SSTRs overexpression (78), especially G1 and G2 stage tumors (79). Indeed, an inverse correlation between SSTR2a expression and NENs differentiation has been observed (80). SSTR2a is particularly over-expressed in PanNETs compared to NENs of different origin (e.g., GI-NENs/NEC). SSTRs represent the molecular target for ^68^Gallium-labeled compounds and PET/CT (^68^ Ga - PET/CT scan) that has recently become the gold standard for the diagnosis and management of these tumors. Recent study by Liverani et al. observed an inverse correlation between ^68^Ga - PET/CT uptake and tumor differentiation in a small GEPNENs subsets (81). Therefore, SSTR2 can be considered for both diagnostic and therapeutic purposes. Intriguingly, SSTR2 is more expressed in primary PanNENs than in metastases (82), suggesting a novel additional role of SSTR2a in monitoring the tumor progression (79). Most of those biomarkers are not yet used in clinical practice. However, multianalyte combinations should show higher sensitivity and might be more effective than the current use of monoanalytes as shown in some studies (83, 84). Several peptides and growth factors have been explored as biomarkers for PanNENs to improve early diagnosis and follow-up of NENs, among these α-Internexin, Paraneoplastic antigen 2 (PNMA2) and X-linked inhibitor of apoptosis (XIAP) are emerging immunocytochemical markers.

**Glucose transporter 1 (GLUT-1)** is a uniporter protein that mediates the transport of glucose molecules through the cell membrane. GLUT-1 is observed to be overexpressed in several tumors, probably related with higher metabolism and cell growth (85). Several studies have shown association between GLUT-1 expression and tumor aggressiveness, poor prognosis and neuroendocrine differentiation in a number of carcinomas (86–88). Fujino, M. et al. investigated the prognostic role of GLUT-1 in G1/G2 PanNENs. GLUT-1 overexpression correlates with grading, Ki-67 mitotic index, vessel invasion, lymph node metastases and poor disease free survival rate (89). In addition, HIF-1α overexpression was observed in GLUT-1 positive cases, suggesting a HIF-1α dependent induction of GLUT-1 in hypoxic conditions (89). In addition, GLUT-1 over expression in NENs correlates with an increased uptake of 2-deoxy-2-[fluorine-18] fluoro-D-glucose and positivity in PET-CT (90). High ^18^F-FDG uptake is a useful prognostic marker in PanNENs (91), thereby GLUT-1 expression may be a good surrogate prognostic marker for ^18^F-FDG captation. Altogether those evidences suggest that GLUT-1 expression might be taken into consideration for PanNENs prognostic assessment. Since ^18^F-FDG uptake by PanNENs is a valuable prognostic marker associated with important aspects of tumor metabolism it is becoming of paramount importance to find biomarkers that correlate with this status for longitudinal analyses in patients. In line with this observation, our preliminary data, presented at the 2019 ESMO meeting reported a prognostic miRNA signature associated with ^18^F-FDG PET status in PanNENs (92).

**Programmed Cell Death Ligand (PD-L1)**, a protein involved in the immune checkpoint, is recently observed to be strongly upregulated in G3 tumor patients both on tumor and infiltrating immune cells, resulting in poor T-cell-mediated tumor surveillance (93). Thus, PD-L1 expression may represent a predictive biomarker for GEP-NENs patients who may benefit from immunotherapy (94). Interestingly, it has been recently reported that DAXX and ATRX molecular alterations correlate with increased tumor-associated macrophage (TAMs) infiltration thereby with inferior Disease Specific Survival rates, suggesting TAMs as potential prognostic biomarkers and targets for immune-modulating therapies in PanNETs (61). Finally, latest publications and communications at international meetings propose novel tissue markers with diagnostic, prognostic and/or therapeutic markers for PanNENs, such as Delta-like protein 3 (DLL-3). Interestingly, PD-L isoform 2 (PD-L2) has been found significantly overexpressed (*p* < 0.001) in PanNENs compared to non-pancreatic NENs (e.g., lung) (95). The same study identified that PD-L2 inversely correlates with presence of tumor necrosis and with PD-L1 expression levels (*p* < 0.03).

**DLL-3** is a member of the Notch ligand family that is aberrantly expressed on the cell surface of Small cell lung cancer (SCLC), Merkel cell Carcinoma (MCC) (96) and other neuroendocrine tumor cells (96–99) making it an attractive therapeutic target in NECs as proposed at latest international conferences, including AACR (96) and ESMO 2019 (100) annual meetings (96) and tested in ongoing trials on SCLC (TAOHE, NCT0306181).

**α-Internexin** is a cytoskeleton protein involved in tumorigenesis and disease progression (101) and is overexpressed in nervous system cell but also in insulinomas (102). Its evaluation in tumor tissue specimens has been observed to be useful as monoanalyte to predict and monitor treatment efficacy in insulinomas (102, 103). Furthermore, combination of α-Internexin and Ki-67 mitotic index, as prognostic multianalytes tests, is observed to predict tumor aggressiveness in insulinomas (89, 104–107). Loss or reduced expression of α-internexin protein represents potential prognostic marker for non-insulinomas PanNENs in terms of overall survival (OS) (102).

**Paraneoplastic antigen 2 (PNMA2)** is a neuronal antigen identified as marker of neurological paraneoplastic syndromes (108). PNMA2 shows correlation with disease progression and recurrence free survival in PanNENs (109).

**X-linked inhibitor of apoptosis (XIAP)** suppresses apoptosis in cancer cells (110, 111). It is a prognostic factor in cancer patients. Despite its role in PanNENs is not well established it is overexpressed in neuroendocrine GI tract and can represent a potential target for therapies (112–114).

Novel forthcoming DNA/RNA markers are also studied. DNA/RNA markers usefulness is mainly explored in the bloodstream via non-invasive liquid biopsy. Nevertheless, detection, analysis, and data interpretation of liquid markers are challenging and still under development. For this reason, many studies explored the expression pattern of DNA/RNA markers and/or molecular mechanisms, such as alternative lengthening of telomeres (ALT), non-coding RNAs, and mutational patterns also and primarily on tumor tissue specimens.

**ALT** is a tissue DNA prognostic marker for NENs. In PanNENs, ALT was shown to correlate with inactivating mutations in ATRX/DAXX genes (115, 116). Despite the literature is controversial about it, ALT expression is associated with larger tumor size, grading, vascular/perineural invasion and metastasis (117, 118). In contrast, other studies have found association with prognosis (119, 120).

**MicroRNAs (miRNAs)** are 21-24 nucleotides non-coding RNAs (ncRNAs) that interfere with gene expression. A plethora of studies have been performed and propose specific tissue miRNA signatures to distinguish PanNENs patients from healthy individuals and the primary tumor from the metastatic disease with a prognostic and/or predictive role. For example, Roldo et al. described a tumor specific miRNA signature defined by miR-103 and miR-107 expression and by the absence of miR-155 expression distinguishing PanNEN from normal pancreatic tissue (121). Furthermore miR-204 is primarily expressed in insulinomas and correlates with insulin expression on tissue (122).

### Genetic Alterations Promoting Nen Development

Before the last decade genetic studies on molecular alterations of GEP-NENs were limited and mainly based on data from genetic syndromes associated with endocrine neoplasms. The diffusion and fruition of next-generation sequencing and other high-throughput techniques (microarray expression, miRNAs, and methylome analyses) in recent years have provided a larger amount of genetic and epigenetic information and a wider view of these malignancies, and especially of PanNENs, from a genetic perspective as reviewed in a very comprehensive manner by several authors (119, 123–130).

This information improved patients' stratification. Indeed, the WHO 2017 update for PanNENs proposed the separation of PanNECs and PanNENs, based on molecular alterations and regardless of the grading (14, 131–133). *TP53* and *RB1* combined loss has been confirmed to be driver mutation of pancreatic carcinoma development. PanNECs represent the 7, 5% of all PanNENs (134) and they are characterized by *TP53* and *RB1* inactivating mutations 20–73 and 71%, respectively while NENs, including G3 NENs with higher Ki-67 percentage and proliferation index do not display these mutations (124, 125, 134–138). RB1 is a key negative regulator of the cell cycle via p16 and other proteins. Indeed, loss of p16 immunostaining has been reported in 20–44% PanNECs, alone or in combination with Rb loss (134, 139–142). Interestingly, RASSF1A, another cell cycle repressor of downstream to Rb displayed methylation of the promoter in 10–60% of PanNECs, pinpointing the crucial role of cell cycle deregulation in carcinomas tumorigenesis (143–146). Interestingly *TP53* inactivation and/or P53 protein nuclear accumulation have been identified in 20-70% and 65-100% of PanNECs respectively (134, 142, 147–149).

A specific mutational pattern has been also reported for PanNENs, that lack *RB/TP53* mutations or an impaired RB/P53 expression. These tumors frequently display *DAXX/ATRXX* (9–25%) and *MEN-1* (10–36%) mutations or protein impaired expression (150, 151). The first whole-exome study on PanNETs, identified *ATRX* and *DAXX* as mutated genes, located in the chromatin remodeling compartment (119). *ATRX/DAXX* loss occurs in 18 and 25% of PanNETs and leads to ALT phenomenon, chromosomal instability and higher tumor stage suggesting this mutation is a late event in the neoplastic transformation (116, 152, 153). A second effect of *ATRX/DAXX* alteration concerns PTEN and, as consequence the inhibition of the PI3K/mTOR pathway (117, 119, 154, 155).

In addition, whole-genome/exome studies identified *PTEN* and *TSC1/2* as potential driver mutations in NENs development when compared to carcinoma tumorigenesis, with a frequency of inactivating lesions among PanNEN cases of 7 and 6%, respectively (119, 156). These alterations, in particular *RB1/TP53* loss, are particularly important for diagnosis and prognosis to distinguish NECs from G3 PanNENs, especially in challenging cases as when morphology and immunostaining are unreliable (131, 132, 151, 157, 158).

### Germline Mutations and Sporadic PanNEN Development

Genetic studies on molecular alteration of GEP-NENs has been limited and mainly based on data from genetic syndromes associated with endocrine neoplasm for a long time. Genetic syndromes with recurrent germline mutated genes such as *MEN, VHL, NF1*, and *TS* (159–164)have been demonstrated to favor GEP-NENs development in about 10% of all NENs (4). Interestingly, somatic mutations on the same genes have been reported to promote sporadic PanNEN onset, with variable frequencies. Data derived from hereditary syndromes first, and from sequencing of sporadic PanNENs later, highlighted the involvement of two main pathways in PanNENs development: cyclin-dependent cell cycle regulation (*MEN-1)* and the PI3K/mTOR pathway (*MEN-1, VHL, NF-1, TS*).

Multiple Endocrine Neoplasia type I is an autosomal dominant disease, promoting the development of pancreatic endocrine tumors in 60% of patients (165). It is caused by germline-inactivating mutations in the *MEN-1* gene (166, 167) and by subsequent somatic loss of the normal allele (168). *MEN-1* gene alteration has been also reported in 44% of sporadic NETs (127). For these reasons it is considered one of the main genes involved in NET biology (119, 156, 169–173). *MEN-1* loss affects a large number of cellular activities, including (a) histone methylation and expression of the *CDKN2C/CDKN1B* cell cycle inhibitors (174); (b) PI3K/mTOR signaling via Akt (175); (c) homologous recombination (HR) through interactions with DNA repair complexes (e.g., RAD51 and BRCA1)(176, 177). In addition, *MEN-1* mutations have been associated with loss of P27 as an early alteration in NET development (178).

Von Hippel–Lindau disease is caused by inactivating mutations of the *VHL* gene. *VHL* is observed to be inactivated also by deletion or methylation in up to 25% of sporadic PanNETs (127). *VHL* inactivation leads to the activation of the hypoxia induced pro-proliferative signaling (179, 180).

Neurofibromatosis type I disease derives from germline mutations of *NF1* that are associated with NEN development in 10% of patients affected by the syndrome. NF1 protein product is a negative regulator of PI3K/mTOR pathway which holds a key role in NEN tumorigenesis (169, 181). Nevertheless, *NF1* has been rarely reported to be mutated in sporadic PanNENs (127).

Inactivating mutations in *TS* lead to Tuberous Sclerosis Complex (TSC) syndrome and to sporadic PanNENs in 35% of cases (127). This is caused by inactivation of *TSC1* and *TSC2*, thus inhibiting PI3K/mTOR signaling downstream of AKT1 (119, 182).

### Chromosomal and Epigenetic Alterations

Mutational events alone cannot be traced back and explain all cases of NEN. Evidence points instead to chromosomal and/or epigenetic alterations as origin of neuroendocrine transformation in about 50% of cases. CNV analysis and whole-genome sequencing (117, 124, 156) allowed the definition of four PanNENs subtypes based on chromosomal alterations: (i) loss of chromosome 11q (where *MEN1* resides); (ii) a recurrent pattern of whole chromosomal loss (RPCL) in association with higher mitotic index, ALT and *ATRX/DAXX* inactivation; (iii and iv) patterns of chromosome gaining, complementary to losses of the RPCL group and associated with higher risk of metastasis (126, 183–187). In addition, whole-genome mutational analysis, identified 10% of germline mutations in base-excision repair (*MUTYH*) and homologous recombination repair (*BRCA2, CHEK2*) genes (119, 182).

From a transcriptional perspective PanNENs have been classified into 3 subtypes, which are related to key pathways of NEN disease, namely, chromatin remodeling in *MEN1*-like tumors, PI3K/mTOR in insulinoma-like tumors and hypoxia-related genes in the metastasis-like primarytumors cluster (188).

DNA methylation alteration is also found and is associated with PanNETs. Hyper-methylation of RASSF1A, HIC-1, CDKN2A, VHL, and MGMT genes for example has been reported in a large fraction of PanNETs (189–191). In contrast, hypo-methylation was reported for ALU and LINE1. In particular LINE1 has been associated to poor prognosis and chromosomal instability in ATRX/DAXX negative tumors (190, 192, 193).

### Liquid Forthcoming Markers in PanNENs

Three key methods allow a comprehensive assessment of the neuroendocrine disease: clinical evaluation, imaging, and biomarkers assessment (62, 84, 194). Imaging is complex, based on sophisticated and expensive technologies, and often fails to predict early changes of the disease and to anticipate progressions or resolve pseudo-progressions (195). In addition, standard serial CT/MRI imaging have well-described sensitivity limitations (196) and may even provide false negative output in comparison to functional imaging ^68^Ga-somatostatin analogs (SSA)-PET/CT (197, 198). Furthermore, imaging can be invasive as it exposes patients to repetitive radiation sessions. Both clinical and imaging strategies, have high intra-observer variability and are operator-dependent (199). In contrast, blood biomarkers represent an easy-to-detect and non-invasive method to evaluate disease with objective measurements (62, 84). The advent of sophisticated and sensitive technologies has revolutionized the concept of biopsy, changing the focus from a tumor tissue-oriented framework to a systemic vision of the disease. Liquid biopsy allows the detection of specific nucleic acids in body fluids and it has particularly benefited from NGS and quantitative PCR approaches, partially overcoming the limit of tumor heterogeneity present in tissue biopsies (195, 200). Application of those analyses to blood samples has clear advantages, by allowing multiple and consecutive measurements to follow disease recurrence and clinical management outcomes. The National Institute of Health (NIH) has classified bio-markers into three categories for diagnosis and/or clinical applications (201): (i) Type 0 markers are ‘indicators of the natural history of disease'. They can directly or indirectly correlate with diagnosis, prognosis, and outcome of the disease. (ii) Type I markers ‘describe the effects of an intervention in accordance with the mechanism of action of the drug' and reflect the general efficacy of treatment through a specific mechanism. Finally, (iii) Type II markers can be used as surrogates for tumor functionality or clinical endpoints (e.g., PFS is often considered for GEP-NENs) (194). In addition, regarding the blood based multianalyte tests (mRNA transcripts, i.e., NETest), the Food and Drug Administration provides guidelines for *in vitro* diagnostic (IVD) tools development. Indeed, FDA defines as IVD “any reagent, instrument, and/or system intended for use in diagnosis of disease or other conditions, including a determination of the state of health, in order to cure, mitigate, treat, or Liquid biomarkers include circulating cell-free DNA (cfDNA), circulating tumor cells (CTCs), small-non-coding molecules, as microRNAs (miRNAs) or long non-coding RNAs (lncRNA), blood transcripts (e.g., NETest) and proteins (Table 4).

**Table 4:** Circulating and tissue molecular biomarkers for PanNENs diagnosis, prognosis and treatment monitoring.

**Molecular Markers**			**Source**	**Level**	**Clinical use**	**References**
Potentially prognostic and/or predictive	Circulating Tumor Cells	CTCs	Serum/plasma	Increased	Related to the PFS and OS	(202)
	Circulating cell free DNA	cfDNA	Serum/plasma	Increased	Indicative of pancreatic tumor origin, correlates with primary tumors mutations (e.g., ATRX/DAXX)	(59)
	Circulating transcripts	NETest	Serum/plasma	Presence of NET “finger print” genes	Prognostic for tumor aggressiveness; predictive for treatment efficacy.	(203)
	MicroRNAs	miRNAs	Serum/plasma*	Up/down—regulated	Diagnostic for site of origin; prognostic and potentially predictive for treatment efficacy.	(58)

PanNENs, Pancreatic Neuroendocrine Neoplasia; PFS, progression-free survival; OS, overall survival. Serum/plasma*: also detected in tumor and healthy tissue. Useful for correlation between circulating and primary tumor markers.

The role of cfDNA in PanNENs is debated. CNV analysis of circulating cfDNA mirrors the presence of tumor-specific genetic alterations of PanNEN cells (59). Nevertheless, the prognostic value of cfDNA harboring *RB1* and *TP53* mutations, typically found in NECs, has not met a consensus yet and it is still under investigation. Similarly genetic alterations affecting *ATRX/DAXX* and *MEN-1* recently found in a subgroup of PanNETs with poor prognosis are not detected yet in cfDNA with a prognostic role (60, 124, 204).

The prognostic significance of CTCs is uncertain and reports about them in NENs are conflicting. Indeed, some studies associate CTCs increase and bone metastasis in NENs (205), whereas others highlight CTCs low sensitivity for PanNENs (84). At present, the 2016 Delphic consensus on circulating biomarkers in NENs has defined CTCs as a non–reliable marker, due to technical limitations in evaluating their number and phenotype.

Circulating miRNAs are more stable than mRNAs in biofluids and are largely explored as prognostic and/or predictive biomarkers in NEN patients (58, 202). Accordingly, several studies have produced signatures of circulating miRNAs associated with PanNEN tissue expression although few reporting prognostic power in PanNENs. Among those miR-21, miR-642, miR-210, miR-196a, miR-96, miR-182, miR-183, and miR-200 are the best characterized (121, 206–208). In addition, a set of 10 miRNAs (miR-125a,−99a,−99b,−125b-1,-342,-130a,−132,−129-2, and−125b-2) has been found to distinguish PanNETs from NEC, whereas miR-204 over-expression resulted to cluster insulinomas (209). Moreover, mir-21 overexpression, which affects PI3K/mTOR pathway via PTEN, has been shown to correlate with higher Ki-67 percentage and liver metastasis in PanNENs (209). Another study reported overexpression of miR-196a as an independent predictor of earlier recurrence, also associated with grade, stage, and lymphatic spread at diagnosis (208). Interestingly, despite the paucity of available preclinical models for NET disease, a metastasis-like (MLP) murine miR-signature (miR-23b,−24-1,−24-2,−27b,−132,-137,−181a1, and−181a2) has been detected and interestingly, it has also found to be overexpressed in about 65% of human PanNETs (188).

LncRNAs can promote angiogenesis, metastasis, and tumor suppressors escape (210–213). The role of lncRNA in PanNENs remains poorly explored in detail yet and most studies investigate their correlation with MEN1 gene-encoding “menin” protein in PanNETs. Modali et al., describe lncRNA *Meg3* (maternally expressed gene) as tumor-suppressor in PanNEN cells. PanNENs which produce Menin can activate *Meg3. Meg3* downregulates *c-Met* affecting cell proliferation, migration and invasion in insulinoma. Indeed, *Meg3* and c-MET levels are described to be inversely correlated, both in MEN1-associated PanNENs and sporadic insulinomas. In a recently published paper, Ji et al. found a significant difference in lncRNA and mRNA expression between pNEN tumors and adjacent normal tissues (214).

### Blood Transcripts (mRNA)—The NETest

The NETest is a PCR-based multianalyte test built on tissue and peripheral blood transcripts using a signature of 51 NETs-related genes (23, 215). This algorithmic multigene assay was designed and validated specifically for GEP and bronchopulmonary NET diseases (83, 203, 216). Recent studies showed that NETest serves as diagnostic tool in PanNENs, since it distinguishes NET disease from cancers of different site of origin or non-neoplastic conditions (e.g., chronic pancreatitis) with 94% accuracy. Indeed, the NETest resulted much more accurate than current validated CgA measurements, which displayed 56% overall accuracy (83).

NETest can act as both type 0 and type II biomarker, as it serves both as diagnostic tool and for prognostication on disease status (stable/progressive disease) and treatment efficacy prediction (154, 203, 216–219). Latest meta-analysis by Oberg et al. recently reported a diagnostic accuracy of NETest of 95–96% with a mean diagnostic odds ratio (DOR) of 5 853, positive likelihood ratio (+LR) of 195, and negative LR of 0.06 in determining the presence of neuroendocrine neoplasia (194). The normalized 51-marker signature is interrogated using 2 separate mathematical algorithmic analyses composing a single score, which is scaled 0–100% (the NETest score). The updated cut-off of NETest score for diagnosis is 20% (220–225). These data are consistent with the definition of IVD functional ability to establish a diagnosis and determine the presence/absence of the disease. In addition, the NETest was 84.5–85.5% accurate as a marker of disease status, distinguishing stable disease from progressive disease at the time of the blood draw (219–221, 224–226). These data show the highest (>80%) concordance with the current Response Evaluation Criteria in Solid Tumors (RECIST) among NET biomarkers, fulfilling NIH proposed cut-off (149). In addition, NETest is observed to be related to functional imaging (e.g., ^68^Ga-somatostatin analogs (SSA)-PET/CT) with 98% concordance in GEP-NETs, including PanNETs (225). Further studies are required to assess whether a blood test can replace imaging for disease monitoring, thus limiting radiation exposure and potential healthcare costs reduction. NETest is also a valuable marker of natural history of the disease (type 0), with an accuracy of 91.5–97.8%. In particular, a cut-off of 40 has been demonstrated to distinguish stable disease ( ≤ 40%) and progressive disease (≥40%) (227). Finally, NETest can be considered also an interventional/response biomarker with 93.7–97.4% accuracy, fulfilling type II biomarker requirements of NIH classification. In particular, a decrease and/or stabilization ( ≤ 40%) of NETest levels correlates with response to PRRT; in contrast with increased levels (≥40%) during therapy and/or follow up which is suggestive of treatment failure (219, 224, 226–228). To enforce NETest clinical value as a PRRT—response biomarker, it can be combined with PRRT Predictive Quotient (PPQ) to improve patient stratification (228). PPQ is a blood-based classifier based on specific variants of the NETest gene signature (encompassing growth factor signaling and metabolomic gene expression) (154, 228–230). PPQ has been demonstrated to predict tumor response to internal radiations in broncopulmonary and GEP-NETs (231). PPQ—positive score can predict PRRT-responders with ~95% accuracy (227). Modlin et al. recently observed that NETest levels significantly decrease after PRRT treatment PPQ positive cohort of “responders,” in contrast with increased level of NETest reported in PPQ-negative cohort of “non-reponders.” NETtest levels negatively correlate with PPQ positivity (*p* < 0.0001) (229, 230). Additionally it has been recenty shown that NETest: (i) high levels (≥40) better predict disease recurrence in post-operative PanNETs alone (AUC: 0.82) or in combination with RECIST criteria (88% accuracy) (232); (ii) is very accurate also for GEP and broncopulmonary NEN with 100% diagnostic accuracy for the latter (233) and (iii) decreased levels after radical resection provide early assessment of surgical efficacy (234).

Very recently, G protein coupled receptor-associated sorting protein-1 (GPRASP-1), known as lysosomal sorting and Beclin2 regulator, has also been proposed as a novel circulating biomarker for neuroendocrine differentiation for PanNENs (235) (Table 5).

**Table 5:** Novel potential biomarkers for PanNENs diagnosis, prognosis and treatment monitoring.

**Putative markers**			**Source**	**Level**	**Clinical use**	**References**
Potentially Prognostic and/or Predictive	Delta-like protein 3	DLL-3	Surgical/endoscopic biopsy	Over-expressed	Potentially prognostic and therapeutic target	(236, 237)
	Tumor-Associated—Macrophages	TAMs	Surgical/endoscopic biopsy	Increased	Associated to reduced DSS	(61)
	G protein coupled receptor-associated sorting protein 1	GPRASP-1	Serum	Down-regulated	Neuroendocrine de-differentiation	(235)
	Glucose transporter 1	GLUT-1	Surgical/endoscopic biopsy	Over-expressed	Prognostic for higher metabolism and tumor aggressiveness	(90)

*PanNENs, Pancreatic Neuroendocrine Neoplasia; GEP-NENs, Gastro-Entero-Pancreatic Neoplasia*.

## Conclusions

Currently available biomarkers for PanNENs have limitations and this unmet need hampers early diagnosis, prognosis and follow-up, stratification of patients for therapy selection and post-operative recurrence identification. Assessment of monoanalytes (e.g., CHGA, SYP) is poorly informative about the pathological status and positivity always need to be supported by further investigations. However, the combination of markers, as CHGA/PP, CHGA/NSE, GLUT-1/Ki-67 have been shown to increase specificity and sensitivity, to trace back to the primary tumor site and to better assess the disease aggressiveness, thus helping clinicians in therapeutic decisions. Liquid biopsy represents the new frontier for PanNEN diagnosis and prognosis, since the sensitivity of technologies is constantly increasing, hence allowing the detection of smaller and smaller amounts of biomarkers with non-invasive procedures. This is leading to earlier diagnosis and more accurate assessment of minimal residual disease after treatment. However, the role of markers such as cfDNA and CTCs is still controversial and requires expensive equipment and well-trained personnel for the analyses. Conversely, the detection of non-coding RNAs, such as miRNAs and lncRNAs is less expensive and more accessible from an economical and a know-how stand-point. Notably, circulating RNAs can not only function as prognostic and/or predictive biomarkers, but also serve as therapeutic targets for tailored approaches, including miRNA replacement. Recently designed clinical trial, SENECA study (NCT03387592) and translational ones as the NET-SEQ study (NCT02586844) and the Royal Marsden PaC-MAn Study (NCT03840460) are at the forefront of this challenge. In particular the Italian SENECA trial focuses on some specific biomarkers on primary tumor tissues and for miRNAs on blood samples while NET-SEQ and PaC-MAN studies are investigating the molecular alterations in intestinal and pancreatic neuroendocrine tumors both in tissue and blood samples. Both studies leverage on NGS sensitivity to discover novel DNA/RNA-based biomarkers from liquid biopsies of NEN patients. We believe those trials will pioneer the identification of the next generation biomarkers for PanNENs.

## Author Contributions

MB wrote the review, prepared figures. FN helped in preparing the figures. SS, AB, and TI provided supervision on the topic. GS edited and commented on the manuscript. IG edited the manuscript. MM conceived and wrote the review.

## Conflict of Interest

The authors declare that the research was conducted in the absence of any commercial or financial relationships that could be construed as a potential conflict of interest.
